# Congenital Diaphragmatic Eventration in an Infant: Diagnostic Pitfalls and Successful Surgical Management

**DOI:** 10.7759/cureus.82017

**Published:** 2025-04-10

**Authors:** Yacine Zouirech, Jawad Bouljrouf, Abir Manni, Monim Ochan, Mounir Kisra

**Affiliations:** 1 Pediatric Surgery Department "A" Children's Hospital, Ibn Sina University Hospital Center, Rabat, MAR; 2 Faculty of Medicine and Pharmacy, Mohammed V University, Rabat, MAR

**Keywords:** congenital anomaly, congenital diaphragmatic eventration, diaphragmatic plication, infant, respiratory distress, thoracotomy

## Abstract

Congenital diaphragmatic eventration (CDE) is a rare condition characterized by abnormal elevation of an intact but thinned diaphragm. Severe cases may mimic congenital diaphragmatic hernia (CDH), leading to respiratory distress in infants. We report the case of a nine-month-old male baby presenting with chronic chest retractions and feeding difficulties, who developed acute respiratory distress initially misdiagnosed as right-sided CDH with pneumonia. Frontal chest radiography suggested herniation, but lateral imaging and surgical findings confirmed right-sided CDE. The patient underwent diaphragmatic plication via a right thoracotomy. Postoperative management, including ventilatory support, led to complete resolution of symptoms and full recovery. This case highlights the importance of accurate imaging, correct diagnosis, and timely surgical intervention.

## Introduction

Congenital diaphragmatic eventration (CDE) is a rare congenital anomaly characterized by elevation of an intact but thinned diaphragm due to muscular deficiency replaced by fibroelastic tissue [1,2]. It accounts for ~5% of all diaphragmatic defects, typically presenting in neonates or infants with a male predominance and possible syndromic associations such as Kabuki or Beckwith-Wiedemann syndromes [1,3]. While asymptomatic cases are often discovered incidentally, severe forms may lead to respiratory distress, feeding difficulties, or recurrent infections [3,4].

Distinguishing CDE from congenital diaphragmatic hernia (CDH) is crucial, as CDH involves a diaphragmatic defect with herniation of abdominal contents, whereas CDE maintains anatomical continuity [1,2]. Diagnosis relies on imaging, including chest radiographs, ultrasound (US), and computed tomography (CT). Lateral sections are particularly useful to differentiate CDE from CDH [1,2,4].

Mild cases may be followed with no surgical intervention, but symptomatic infants benefit from diaphragmatic plication to restore lung function and prevent complications [5,6]. Early intervention, especially through thoracoscopic approaches, has been shown to significantly improve outcomes and quality of life for affected infants [1,5].

This case underscores the diagnostic challenges of CDE and emphasizes the integration of clinical and imaging data for timely surgical management.

## Case presentation

A nine-month-old male baby was admitted in January 2025 for evaluation of suspected diaphragmatic hernia. He had progressive chest retractions and feeding difficulties since one month of age. There was no history of first-degree marriage.

He was born at term after an uneventful pregnancy and vaginal delivery. He was exclusively breastfed and began solid foods at five months. Vaccinations were stopped at four months due to recurrent illness. In November 2024, he was hospitalized for respiratory distress. A frontal chest X-ray suggested right diaphragmatic hernia, and surgery was deferred.

In late December 2024, he developed a 10-day productive cough and rhinorrhea, progressing to respiratory distress by December 28. He was admitted to the ICU on December 30 with presumed pneumonia and suspected right diaphragmatic hernia. Treated with ceftriaxone at a pediatric dose and nebulized salbutamol, he was stabilized by January 6, prompting referral for surgical evaluation.

On admission, he was alert and reactive, weighing 10 kg, measuring 70 cm, with stable vital signs: heart rate (HR) 125 bpm, respiratory rate (RR) 38/min, peripheral oxygen saturation (SpO₂) 95% on room air, and temperature 36.8°C. Examination revealed bilateral symmetric intercostal and subcostal retractions, mild expiratory wheezing, and diminished right basal breath sounds. No cyanosis, clubbing, or thoracic deformities were noted. Cardiac and abdominal exams were unremarkable.

**Figure 1:**
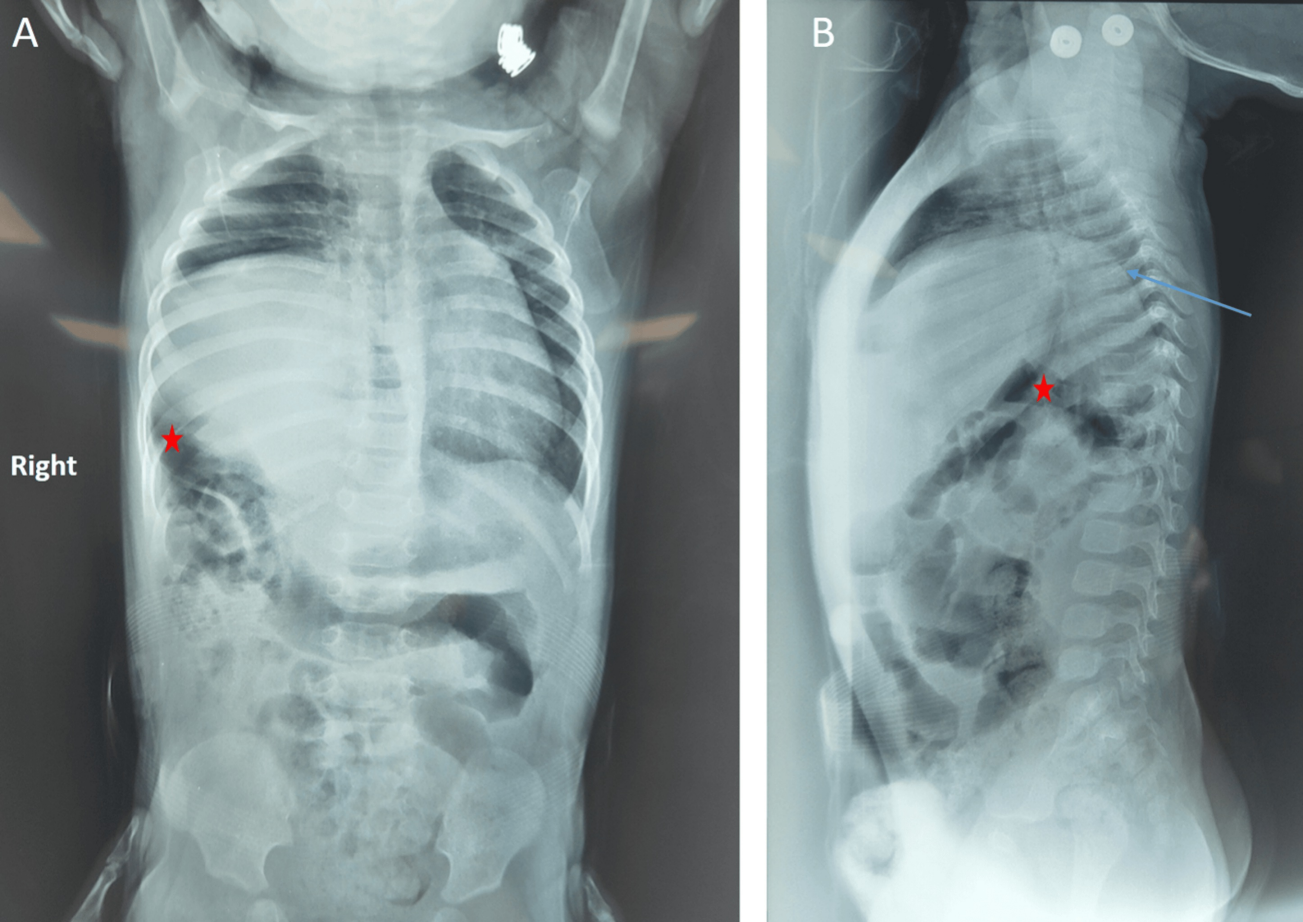
Frontal (A) and lateral (B) chest radiographs of the patient (A) The frontal view shows right-sided lucencies (red star) with mediastinal shift, initially suggestive of a congenital diaphragmatic hernia. (B) The lateral view reveals elevation of the right hemidiaphragm (blue arrow) with intrathoracic bowel loops (red star), consistent with congenital diaphragmatic eventration.

Frontal chest X-ray showed right-sided lucencies with mediastinal shift, initially suggestive of a CDH (Figure 1A), while the lateral view revealed an elevated right hemidiaphragm with intrathoracic bowel loops (Figure 1B), consistent with CDE.

**Figure 2:**
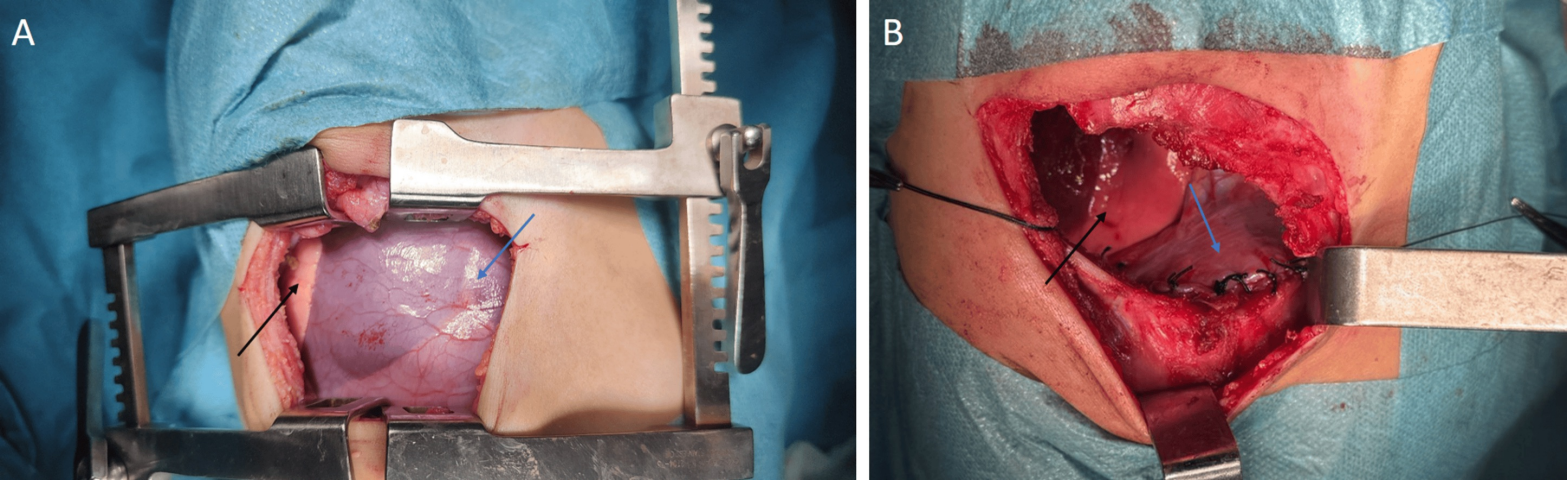
Intraoperative findings before and after diaphragmatic plication (A) Intraoperative view showing a thinned, elevated right hemidiaphragm (blue arrow) without herniation, confirming congenital diaphragmatic eventration. The underlying lung is visible and compressed (black arrow). (B) Completed plication with non-absorbable interrupted sutures (blue arrow) restoring diaphragmatic tension and position. The re-expanded lung is also visible (black arrow).

Once the patient maintained adequate oxygenation in room air, he underwent surgical exploration via a right posterolateral thoracotomy through the sixth intercostal space. Intraoperative findings confirmed a thinned, elevated right hemidiaphragm without herniation, and no evidence of lung hypoplasia was found (Figure 2A)*. *Diaphragmatic plication was performed using non-absorbable interrupted sutures to reinforce tension and restore anatomical position, followed by chest tube placement (Figure 2B).

**Figure 3:**
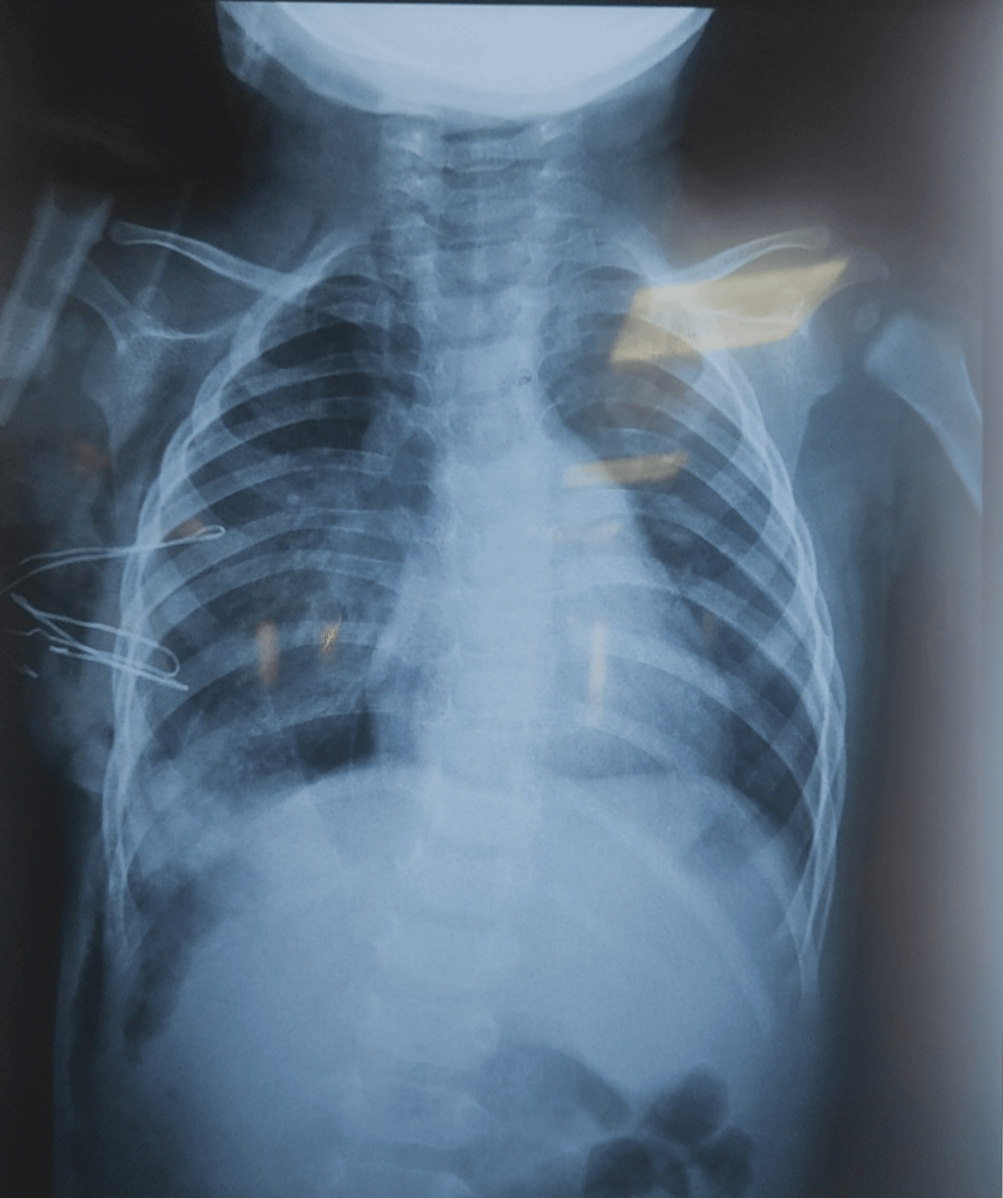
Postoperative chest X-ray Frontal chest radiograph showing corrected right hemidiaphragm position after plication with partial lung re-expansion and a mild right pneumothorax. The chest tube is visible in situ.

Postoperatively, the patient was closely monitored for 48 hours in the surgical ICU for potential complications, including ventilatory support and management of any intra-abdominal pressure. A chest X-ray on postoperative day 1 showed a corrected right hemidiaphragm position after plication with partial lung re-expansion and a mild right pneumothorax. The chest tube was removed on day 2 (Figure 3). He resumed oral feeding, completed a seven-day course of IV antibiotics, and was discharged on postoperative day 7 with resolved retractions. At one-month follow-up, he remained asymptomatic with normal diaphragm position and weight gain. Outpatient monitoring was ongoing.

## Discussion

CDE presents diagnostic difficulties due to its radiographic resemblance to CDH. In this case, frontal chest radiography initially suggested CDH, but lateral imaging and surgical exploration confirmed right-sided CDE. This highlights the need for multi-view imaging, including lateral X-rays, US, or CT, to ensure diagnostic accuracy and avoid unnecessary surgical procedures [1,2,4]. Misdiagnosis of CDE can lead to inappropriate surgical planning, such as unnecessary abdominal exploration, as seen in several cases where CDE was initially mistaken for CDH [1,6].

CDE arises from a congenital deficiency of diaphragmatic muscle replaced by fibroelastic tissue, preserving anatomical continuity and thus differing from CDH [1]. Although earlier studies suggested left-sided predominance, recent series report more frequent right-sided involvement in neonates, possibly due to structural support from the heart on the left [3,4].

Clinical presentations vary widely. Asymptomatic infants may be diagnosed incidentally, while symptomatic cases exhibit tachypnea, retractions, recurrent infections, or feeding intolerance [4,5]. Imaging is pivotal for diagnosis; lateral views are especially useful when CDH is suspected. Diaphragmatic US can confirm paradoxical movement of the eventrated diaphragm [2].

Surgical diaphragmatic plication remains the gold standard for symptomatic cases, as it flattens the diaphragm and improves lung expansion [6,7]. Minimally invasive approaches such as thoracoscopy or laparoscopy are increasingly used due to better cosmetic and postoperative outcomes, though open thoracotomy remains suitable for unstable patients or uncertain diagnoses [6,7]. In our patient, prompt surgical repair led to complete resolution of respiratory symptoms. This mirrors outcomes reported in the literature, where over 90% of patients show improved breathing and feeding postoperatively [3,5,7].

Postoperative complications such as pneumothorax or effusion are rare and typically managed conservatively [2,5]. Long-term follow-up is crucial to monitor lung development and recurrence [4,5].

## Conclusions

CDE, although rare and often asymptomatic, may present with significant respiratory and digestive symptoms, sometimes mimicking more severe anomalies such as CDH. Accurate diagnosis requires a high index of suspicion, supported by detailed imaging studies such as chest radiographs, US, and CT. Recent advancements in diagnostic modalities have further improved the accuracy of diagnosis and management strategies.

Surgical plication remains the gold standard in treating symptomatic infants, with excellent outcomes when performed promptly. Early recognition and appropriate intervention are critical in preventing long-term pulmonary complications and ensuring optimal growth and development. Newer surgical techniques, including minimally invasive approaches, have further enhanced patient recovery and reduced postoperative morbidity. Ongoing follow-up is essential for monitoring lung development and detecting any potential recurrence.
